# A 50+ Sunscreen Containing *Polypodium Leucotomos* Extract, Ellagic Acid, and Niacinamide in Facial Melasma and Photoaging‐Related Hyperpigmentation: A Pilot Observational Study Using Line‐Field Confocal Optical Coherence Tomography

**DOI:** 10.1111/jocd.70732

**Published:** 2026-02-08

**Authors:** Alessia Villani, Lucia Genco, Luigi Coronella, Valentina Ventura, Massimo Milani, Stefano Alfano, Francesca Colombo

**Affiliations:** ^1^ Dermatologia Azienda Ospedaliero Universitaria Federico II –Napoli Naples Italy; ^2^ Cantabria Labs Difa Cooper MD Caronno Pertusella Italy

**Keywords:** confocal optical coherence tomography, ellagic acid, melasma, niacinamide, *Polypodium leucotomos*, sunscreen

## Abstract

**Introduction:**

Melasma is an acquired facial hyperpigmentation disorder that disproportionately affects sun‐exposed areas. Current treatments show limited efficacy and tolerability.

**Aim:**

To assess, in a non‐controlled prospective pilot trial, the efficacy of a fluid cream SPF 50+ sunscreen containing ellagic acid, niacinamide, and a non‐UV‐filtering extract of *Polypodium leucotomos* (PLE).

**Methods:**

Twenty women (mean age 40 ± 10 years) with melasma or sun‐induced hyperpigmentation applied the sunscreen every morning and every 2 h during sun exposure for 12 weeks. The primary endpoint was the change in Melasma Area and Severity Index (MASI) at week 12 and week 24 (follow‐up). The secondary endpoints were Line‐field Confocal Optical Coherence Tomography (LC‐OCT) evaluation of pigment distribution and hyper‐reflective cells. Local tolerability was also assessed.

**Results:**

All subjects completed the study. Baseline MASI (6.9 ± 3.2) decreased to 4.4 ± 3.3 at week 12 and 3.9 ± 3.1 at week 24 (−44% from baseline, *p* = 0.0001). LC‐OCT pigment scores declined from 2.2 ± 0.8 to 1.5 ± 0.6 at week 24 (*p* = 0.04), and hyper‐reflective cells decreased from 47% to 26%. The product was well tolerated, with tolerability scores near to zero.

**Conclusion:**

This pilot proof‐of‐concept study suggests that an SPF 50+ sunscreen containing ellagic acid, niacinamide, and PLE may reduce melasma severity and improve hyperpigmentation with excellent tolerability. These findings warrant confirmation in larger controlled trials.

**Trial Registration:**

clinical registration number ISRCTN18053239

## 1 Introduction

Melasma is a common, multifactorial acquired skin disorder characterized by symmetric brown patches most often found on sun‐exposed areas of the face [1]. Among the factors that contribute to Melasma can be distinguished external factors, such as ultraviolet (UV) radiation exposure, and hormonal factors (including sex hormones, pregnancy, and oral contraceptives), as well as genetic predisposition and skin inflammation [2]. Recent research highlights the association of melasma and photoaging, involving not just melanocytes but also changes in the dermis, basement membrane (BM), vascularization, and mast cell activity [3]. In particular, the pathogenesis of melasma involves chronic exposure to UV and visible light as a primary trigger [4]. UV radiation upregulates melanogenic pathways, increasing oxidative stress and causing structural DNA damage at the level of the skin [5]. This process leads to increased melanin production and persistent hyperpigmentation. Damage and discontinuity of the basement membrane, a structure that separates the epidermis from the dermis, is frequently observed in Melasma patients [6]. In particular, the disruption of this membrane facilitates the migration of melanocytes into the dermis, contributing to the chronicity and resistance to treatment [7]. In addition, the disruption of BM is often linked to inflammation and oxidative stress [8]. Melasma treatment remains challenging due to high recurrence rates and the need for long‐term, multi‐modal management [9]. The first line therapy for melasma typically includes topical agents, such as hydroquinone and triple combination creams containing hydroquinone, tretinoin, and corticosteroids [10]. Other treatment options include non‐hydroquinone depigmenting agents, oral tranexamic acid, chemical peels, microneedling, and laser/light‐based therapies, with combination therapies (e.g., topical plus procedural) having better results than monotherapies [11]. Despite all the treatment options we discussed, Melasma remains a condition with high recurrence rates of relapse [12]. After topical therapies, recurrence of melasma is common, especially after discontinuation. When combined, topical therapy and oral tranexamic acid demonstrate a reduction in the recurrence rate of melasma (e.g., 18% vs. 64% at 24 weeks), but side effects and relapse after stopping are concerns [13].

Photoprotection remains a cornerstone in both the treatment and prevention of melasma [14]. The use of rigorous photoprotection blocks the trigger action of UV and visible light, directly reducing new pigment formation and helping to fade existing hyperpigmentation [15]. After successful treatment with topical agents or procedures, ongoing photoprotection is necessary to maintain results and prevent relapses. The different composition of a sunscreen grants efficacy against different factors that can influence the development of melasma. Photoprotective molecules such as iron oxides and *Polypodium leucotomos* extract, prevent UV and visible light from stimulating melanocytes, reducing new pigment formation and relapses [16]. Some commercially available sunscreens contain topical depigmenting agents that act synergistically in reducing skin hyperpigmentation, improving the quality of life of patients [17]. Considering data available in the literature, this clinical trial aims to evaluate the efficacy of a commercially available broad‐spectrum sunscreen (Heliocare 360 Pigment Solution Fluid) containing Iron oxide, Polypodium Leucotomos Leaf Extract, Ellagic acid, and Niacinamide in reducing the signs of melasma in Caucasian subjects.

## 2 Study Aim

The study aimed to evaluate the efficacy of a fluid emulsion with SPF 50+ containing ellagic acid, niacinamide, Fernblock + (*Polypodium leucotomos* extract), and vitamin E (Heliocare 360 Pigment Solution Fluid SPF 50+, Cantabria Labs Difa Cooper) in the prevention and treatment of melasma and photoaging, using in vivo confocal optical coherence tomography.

## 3 Materials and Methods

### 3.1 Study Design

This was a pilot, prospective, open‐label study with a total duration of 3 months, followed by a three‐month follow‐up. The study was conducted between February 2023 and July 2025, in accordance with Good Clinical Practices procedures and the Helsinki Declaration and consistent with the Good Clinical Practice (GCP) regulatory requirements. All subjects provided a signed informed consent. The study protocol was approved by an external Investigational Review Board in January 2023 (DF‐01‐HCPS 10/01/2023). The clinical trial registration number is ISRCTN18053239. A total of 20 female (mean age 40 ± 10) with facial melasma and photoaging meeting all inclusion/exclusion criteria were enrolled in a 6‐month trial, after their written informed consent. Eligible subjects were Caucasian women aged 25 to 60 years with facial melasma present for at least 5 months. The main exclusion criteria included the presence of other facial disorders, the use of topical treatments within the 2 months preceding study initiation, and pregnancy or lactation. Patients were instructed to apply the product every morning and reapply it every 2 h during sun exposure, for a period of 12 weeks. The study lasted 3 months, followed by a three‐month follow‐up phase. Participants attended three study visits: at baseline (T0), after 12 weeks of treatment (T1), and after 24 weeks for the follow‐up evaluation (T2).

### 3.2 Study Outcomes

The primary outcome was the evaluation of clinical efficacy through the Melasma Area Severity Index (MASI) [18]. The MASI was calculated by evaluating three factors: area involved (A), darkness (D), and homogeneity (H), across four distinct regions: the forehead, right malar region, left malar region, and chin. The total score range is 0 to 48. Secondary outcomes included the parameters assessed by in vivo Line‐field Confocal Optical Coherence Tomography (LC‐OCT), a non‐invasive technique for detecting abnormal pigment deposits, providing additional cytological information useful for the diagnosis, classification, and monitoring of melasma [19]. High‐resolution in vivo imaging was performed using a Line‐field Confocal Optical Coherence Tomography (LC‐OCT) device (DeepLive, DAMAE Medical, Paris, France), which combines the principles of confocal microscopy and optical coherence tomography to provide real‐time cross‐sectional and en face views of the skin with quasi‐histological resolution. The device utilizes a supercontinuum laser source (λ = 800 ± 40 nm) with an axial resolution of approximately 1.1 μm and a lateral resolution of 1.3 μm, enabling precise visualization of both epidermal and superficial dermal structures to a depth of 500 μm. LC‐OCT imaging was performed on a representative hyperpigmented lesion located on the forehead, right malar region, left malar region, and chin region for each subject. The skin was gently cleansed, and a thin layer of immersion gel was applied before placing the objective lens in contact with the skin surface. Vertical (B‐scan) and horizontal (en face) acquisitions were obtained at baseline (T0), after 12 weeks of treatment (T1), and at the 24‐week follow‐up (T2). Each image was analyzed by two independent blinded dermatologists with expertise in LC‐OCT interpretation. LC‐OCT evaluation included: (1) pigment localization at the epidermis, dermal–epidermal junction, and superficial dermis using a qualitative 0–3 hyperpigmentation scale, and (2) the percentage of hyperreflective basal keratinocytes around dermal papillae was quantified using manual segmentation and expressed as the mean value of three independent measurements per site. All LC‐OCT data were analyzed using the manufacturer's software (DeepLive Analysis Suite v2.1, DAMAE Medical). Local tolerability (erythema, xerosis, burning, itching) was assessed on a 0–3 scale (0 = absent, 3 = severe).

### 3.3 Statistical Analysis

In view of the proof‐of‐concept nature of the present trial, a formal sample size calculation was not performed. We decided to enroll at least 20 evaluable subjects. Statistical analyses were conducted using GraphPad statistical software version 9.0 (GraphPad Software Inc., La Jolla, CA, USA). A non‐parametric test (Wilcoxon signed rank test) was used to compare data at baseline and at the end of treatment. Confidence intervals 95% (CI) were calculated for the absolute difference of MASI. Data are expressed as mean ± standard deviation (SD), and a *p* < 0.05 was considered significant.

## 4 Results

**FIGURE 1:**
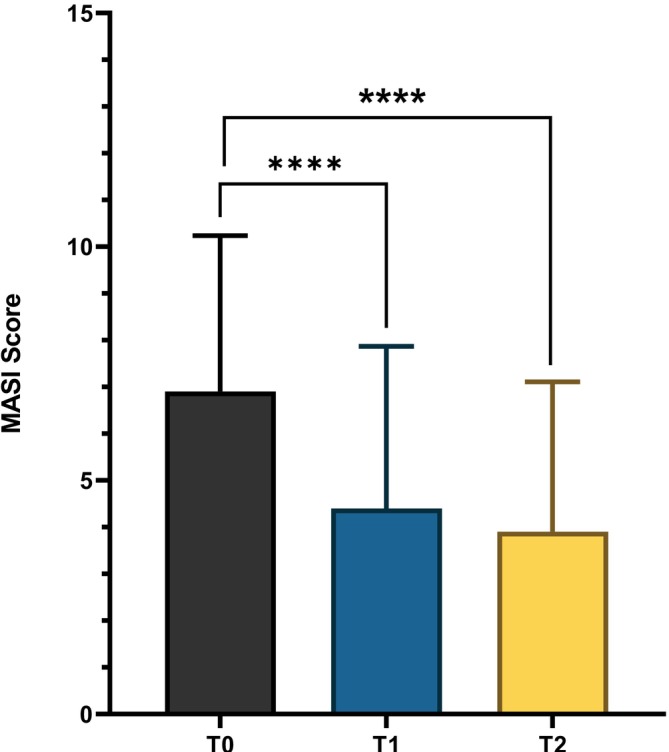
MASI score evaluated at baseline (T0), after 12 weeks of treatment (T1), and after 24 weeks at the follow‐up visit (T2). ****p < 0.0001.

A total of 20 women (mean age 40 ± 10 years) with facial melasma and photoaging were enrolled, and all subjects completed the treatment period. The main hyperpigmented lesions were located on the forehead (*n* = 13), cheek (*n* = 4), and jaw (*n* = 3). At baseline, the mean Melasma Area and Severity Index (MASI) score was 6.9 ± 3.3. After 12 weeks of treatment, the MASI score significantly decreased to 4.4 ± 3.4 (absolute difference−2.5; 95% CI: −3.1, −1.9; 36% reduction, *p* < 0.0001). This improvement was maintained at the 24‐week follow‐up, with a further significant reduction in MASI score to 3.9 ± 3.1 (absolute difference vs. baseline −3.0; 95% CI: −3.6, −2.4; 44% reduction *p* < 0.0001; Figure 1).

**FIGURE 2:**
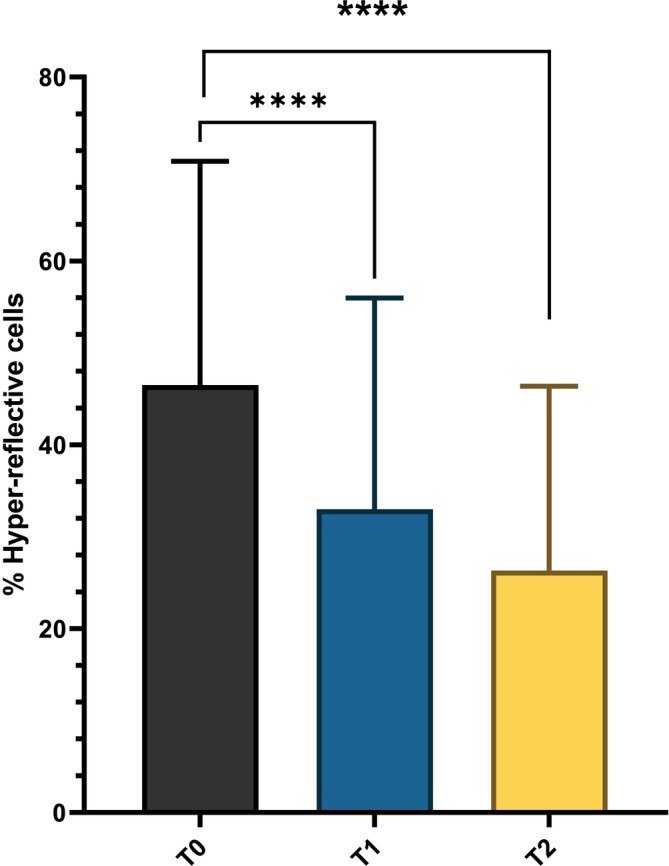
Percentage of Hyper‐reflective cells at baseline (T0), after 12 weeks of treatment (T1), and after 24 weeks at the follow‐up visit (T2). ****p < 0.0001.

Hyper‐reflective cells observed with LC‐OCT correspond to hyperpigmented basal keratinocytes, which can be reduced through treatment. At baseline (T0), the proportion of hyper‐reflective cells was 47% ± 24%. After 12 weeks of treatment, this value significantly decreased to 33% ± 22% (*p* < 0.0001), with a further significant reduction observed at the 24‐week follow‐up (26% ± 20%, *p* < 0.0001, Figure 2).

**FIGURE 3:**
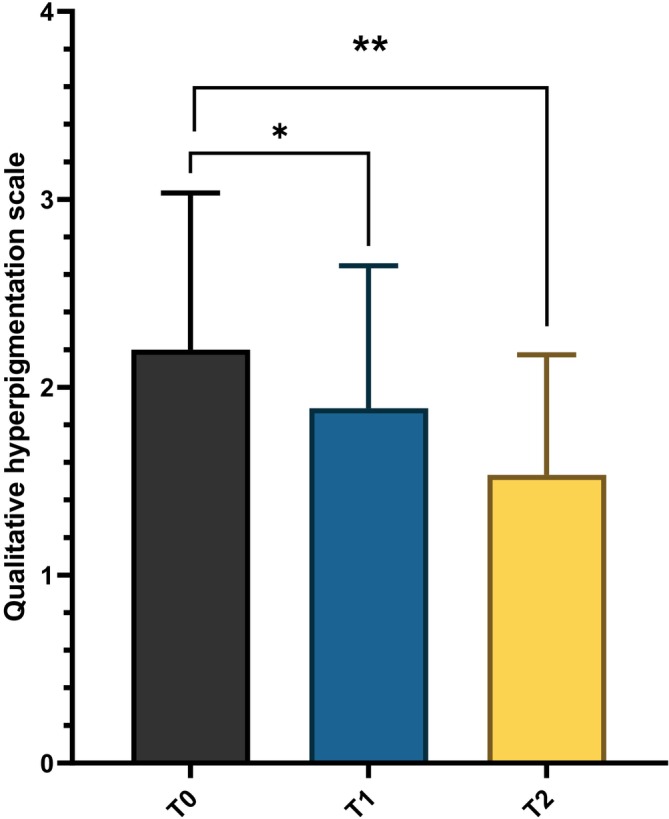
Qualitative hyperpigmentation scale evaluated at baseline (T0), after 12 weeks of treatment (T1), and after 24 weeks at the follow‐up visit (T2). *p < 0.05, **p < 0.01.

**FIGURE 4:**
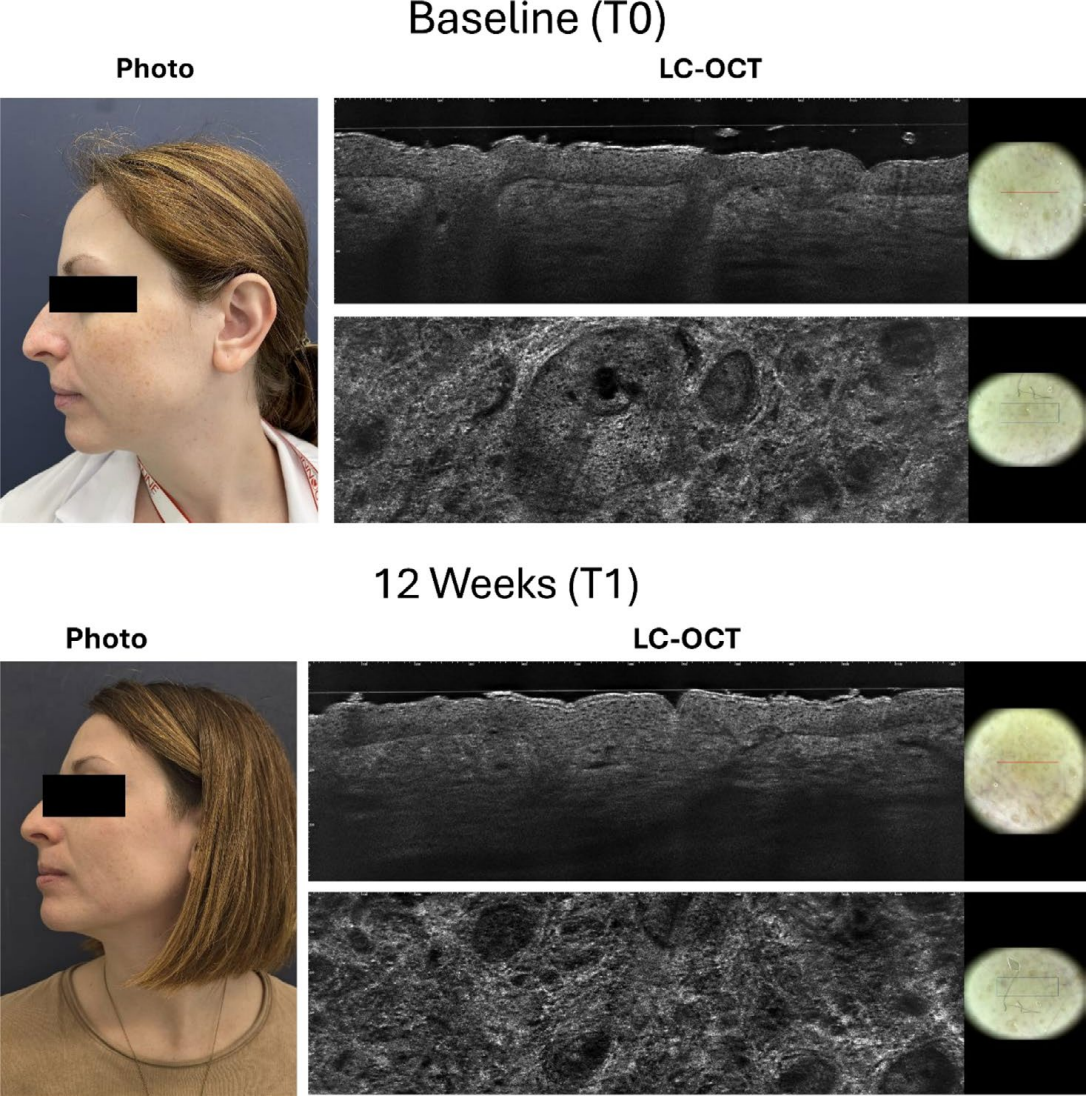
Clinical and LC‐OCT results of a representative patient taken at baseline (T0) and after 12 weeks of treatment (T1).

The pigmentation was monitored during the study using a qualitative hyperpigmentation scale with a baseline value of 2.2 ± 0.8. The score was reduced to 1.9 ± 0.7 after 12 weeks of treatment (*p* < 0.05), with a further significant reduction observed at the 24‐week follow‐up (1.5 ± 0.6, *p* = 0.002, Figure 3). Figure 4 shows the clinical and LC‐OCT results of a representative patient taken at baseline (T0) and after 12 weeks of treatment (T1). Clinically, a visible reduction in the intensity and extension of hyperpigmented macules was observed after treatment (T1), with a more uniform skin tone and decreased contrast between lesional and perilesional areas. LC‐OCT imaging at baseline (T0) showed marked hyperreflectivity at the basal epidermal layer, corresponding to clusters of hyperpigmented basal keratinocytes and an irregular pigment distribution along the dermal–epidermal junction. After 12 weeks (T1), a substantial decrease in hyperreflective cells and a more homogeneous epidermal reflectivity pattern were evident, indicating reduced pigmentation and improved epidermal architectural regularity. The product was well tolerated: tolerability scores averaged 0.3 at week 12 and 0 at week 24.

## 5 Discussion

This pilot proof‐of‐concept study suggests efficacy and very good tolerability of a 50+ sunscreen containing ellagic acid, niacinamide, and the non‐filtering sun protective substance PLE in the treatment of sun‐induced facial melasma‐hyperpigmentation.

Melasma is a chronic, acquired hyperpigmentary disorder characterized by symmetric, brownish hyperpigmented patches, most commonly affecting sun‐exposed areas [20]. It predominantly occurs in women, especially those with darker skin types. The condition can significantly impact the quality of life due to its visible appearance and frequent recurrences [21]. Although various therapeutic approaches have been proposed for melasma, no definitive treatment has yet been established as the gold standard [22]. In addition to hydroquinone, commonly used options include retinoic acid, kojic acid, azelaic acid, and several chemical peels such as lactic, salicylic, glycolic, and trichloroacetic acids [23]. Physical interventions, including dermabrasion and laser therapy, have also been investigated, but with limited success and common relapses [24]. Broad‐spectrum sunscreen remains at the base and is essential to prevent and treat hyperpigmentation and relapses of melasma. In this pilot proof‐of‐concept study, we investigated a topical product with SPF 50+ containing Fernblock (*Polypodium leucotomos* extract), niacinamide, vitamin E, and ellagic acid as a potential strategy for the prevention and treatment of melasma and photoaging, using in vivo Line‐field Confocal Optical Coherence Tomography. Fernblock is an extract of *Polypodium leucotomos* (PLE), a tropical fern native to Central and South America [25]. Over the past three decades, both clinical and non‐clinical studies have demonstrated its protective effects against ultraviolet (UV) radiation damage, contributing to the reduction of photoaging manifestations, including UV‐induced pigmentation [26]. The extract contains phenolic compounds with potent antioxidant activity that mitigate UV‐induced oxidative stress [27]. In addition, PLE exhibits anti‐inflammatory effects, further supporting its role in photoprotection. Clinical evidence has also supported its use in melasma: in a randomized, placebo‐controlled trial of women aged 18–50 years with epidermal melasma, oral administration of Fernblock twice daily for 12 weeks led to a significant reduction in Melasma Area and Severity Index (MASI) scores (from 5.7 to 3.3; −42%, *p* < 0.05) [28]. The antioxidant activity of Fernblock may be potentiated by vitamin E, also contained in the tested product, which enhances radical scavenging capacity. Niacinamide is widely recognized for its anti‐inflammatory properties and its ability to reinforce the skin barrier [29]. Moreover, it inhibits the transfer of melanosomes from melanocytes to keratinocytes, thereby reducing melanin distribution in the upper epidermis and diminishing visible hyperpigmentation [30]. Ellagic acid primarily acts by chelating copper ions at the active site of tyrosinase, a key enzyme in melanogenesis, thereby decreasing its catalytic activity and reducing melanin synthesis [31]. Additionally, ellagic acid modulates several signaling pathways involved in melanogenesis. Considering the multifactorial pathogenesis of melasma, a comprehensive, combination‐based treatment strategy employing an SPF 50+ formulation enriched with antioxidant, anti‐inflammatory, and melanin‐modulating agents may effectively target multiple pathogenic pathways, thereby enhancing therapeutic efficacy. This pilot proof‐of‐concept study suggested that the tested SPF 50+ sunscreen containing ellagic acid, niacinamide, and *Polypodium leucotomos* extract may reduce melasma severity and improve sun‐induced hyperpigmentation, with excellent local tolerability. Thirty‐five percent of patients achieved an excellent clinical response rate (> 50% MASI score reduction), 45% demonstrated a good response rate (25%–50% MASI score reduction), and only 20% showed a modest response rate (< 25% MASI reduction). After 12 weeks of sunscreen application, the MASI score decreased by 36%, and at week 24 (the end of follow‐up), it showed a further reduction of 44% from baseline. The reduction in MASI scores (44% at week 24) exceeded the calculated Minimal Clinically Important Difference (MCID) of 1.65 (calculated using a distribution‐based approach that pooled the standard deviations associated with the baseline mean [32]), supporting the clinical relevance of the observations. The inclusion of LC‐OCT added an objective dimension, showing reduced epidermal pigmentation. Several limitations should be considered in evaluating our study results. This was not a double‐blind trial, and no control group was used. However, the present study was a pilot proof‐of‐concept trial, and to increase the internal validity of our results, we employed objective instruments (LC‐OCT) to assess patients' skin, which enhances the reliability of the findings by reducing potential operator error or subjective bias. As an open‐label, non‐controlled trial, improvements cannot be definitively attributed to the active ingredients alone, as photoprotection itself is therapeutic. The small sample size and exclusive inclusion of Caucasian women limit generalizability to darker phototypes. Furthermore, seasonal UV variations and individual sun habits were not controlled, which may have influenced at least in part the results. Further, larger prospective controlled studies using Fernblock technology are needed to confirm whether this therapeutic strategy could help manage melasma. Our results could be relevant for future large, controlled studies by providing useful data for hypothesis generation and correct sample size calculation.

## 6 Conclusions

Sunscreen remains vital for melasma management. This pilot proof‐of‐concept study indicates that a formulation containing PLE, ellagic acid, and niacinamide is well‐tolerated and may offer potential benefits as an adjunct strategy in reducing hyperpigmentation. These preliminary findings suggest that further investigation in randomized, vehicle‐controlled trials with diverse populations is warranted. Furthermore, these preliminary findings may help inform sample size estimation and design of future controlled clinical trials.

## Author Contributions

All authors participated in writing, review and editing. All authors contributed to the review and final approval of the manuscript. All authors have read and agreed to the published version of the manuscript.

## Funding

The present trial was supported by an unrestricted grant by Cantabria Labs Difa Cooper. Institutional Review Board Statement: The study protocol was approved by an external Investigational Review Board in January 2023 (DF‐01‐HCPS 10/01/2023). The clinical trial registration number is ISRCTN18053239.

## Consent

All participants provided written informed consent and a photo consent statement before starting the study. Written informed consent has been obtained from the patients to publish this paper.

## Conflicts of Interest

Massimo Milani, Stefano Alfano and FA are employees of Cantabria Labs Difa Cooper. All other authors declare no conflicts of interest.

## Data Availability

The data that support the findings of this study are available on request from the corresponding author. The data are not publicly available due to privacy or ethical restrictions.

## References

[1] O. A. Ogbechie‐Godec and N. Elbuluk , “Melasma: An up‐To‐Date Comprehensive Review,” Dermatology and Therapy 7, no. 3 (2017): 305–318, 10.1007/s13555-017-0194-1. 28726212 PMC5574745

[2] S. H. Kwon , Y. J. Hwang , S. K. Lee , and K. C. Park , “Heterogeneous Pathology of Melasma and Its Clinical Implications,” International Journal of Molecular Sciences 17, no. 6 (2016): 824, 10.3390/ijms17060824. 27240341 PMC4926358

[3] A. C. C. Espósito , D. P. Cassiano , C. N. da Silva , et al., “Update on Melasma—Part I: Pathogenesis,” Dermatology and Therapy 12, no. 9 (2022): 1967–1988, 10.1007/s13555-022-00779-x. 35904706 PMC9464278

[4] J. Yang , J. Zeng , and J. Lu , “Mechanisms of Ultraviolet‐Induced Melasma Formation: A Review,” Journal of Dermatology 49, no. 12 (2022): 1201–1210, 10.1111/1346-8138.1654. 35946331

[5] R. Khanna , A. Nowah , D. Morris , and S. R. Desai , “Pathogenesis of Melasma,” Dermatological Reviews 4, no. 1 (2023): 12–16.

[6] D. Gu , R. Pan , X. Meng , et al., “What Lies Behind Melasma: A Review of the Related Skin Microenvironment,” International Journal of Dermatology 64, no. 2 (2024): 256–265. 39212112 10.1111/ijd.17453

[7] S. Katiyar and D. Yadav , “Correlation of Oxidative Stress With Melasma: An Overview,” Current Pharmaceutical Design 28, no. 3 (2022): 225–231. 34736377 10.2174/1381612827666211104154928

[8] B. Torres‐Álvarez , I. G. Mesa‐Garza , J. P. Castanedo‐Cázares , et al., “Histochemical and Immunohistochemical Study in Melasma: Evidence of Damage in the Basal Membrane,” American Journal of Dermatopathology 33, no. 3 (2011): 291–295. 21317614 10.1097/DAD.0b013e3181ef2d45

[9] P. R. Cohen , “Comment on: Facial Hyperpigmentation in Skin of Color: Special Considerations and Treatment,” American Journal of Clinical Dermatology 18, no. 4 (2017): 593–594. 28597179 10.1007/s40257-017-0303-z

[10] J. McKesey , A. Tovar‐Garza , and A. G. Pandya , “Melasma Treatment: An Evidence‐Based Review,” American Journal of Clinical Dermatology 21, no. 2 (2020): 173–225. 31802394 10.1007/s40257-019-00488-w

[11] C. Gan and M. Rodrigues , “An Update on New and Existing Treatments for the Management of Melasma,” American Journal of Clinical Dermatology 25, no. 5 (2024): 717–733. 38896402 10.1007/s40257-024-00863-2 PMC11358250

[12] D. P. Cassiano , A. C. C. Espósito , C. N. da Silva , et al., “Update on Melasma—Part II: Treatment,” Dermatology and Therapy 12, no. 9 (2022): 1989–2012. 35906506 10.1007/s13555-022-00780-4 PMC9464276

[13] Z. Piętowska , D. Nowicka , and J. C. Szepietowski , “Understanding Melasma‐How Can Pharmacology and Cosmetology Procedures and Prevention Help to Achieve Optimal Treatment Results? A Narrative Review,” International Journal of Environmental Research and Public Health 19, no. 19 (2022): 12084. 36231404 10.3390/ijerph191912084 PMC9564742

[14] D. Morgado‐Carrasco , J. Piquero‐Casals , C. Granger , C. Trullàs , and T. Passeron , “Melasma: The Need for Tailored Photoprotection to Improve Clinical Outcomes,” Photodermatology, Photoimmunology & Photomedicine 38, no. 6 (2022): 515–521. 10.1111/phpp.12783 PMC9790748 35229368

[15] J. Ocampo‐Candiani , R. Alas‐Carbajal , J. F. Bonifaz‐Araujo , et al., “Latin American Consensus on the Treatment of Melasma,” International Journal of Dermatology 64, no. 3 (2025): 499–512. 39415312 10.1111/ijd.17522 PMC11840225

[16] C. Parrado , M. Mascaraque , Y. Gilaberte , A. Juarranz , and S. Gonzalez , “Fernblock (Polypodium Leucotomos Extract): Molecular Mechanisms and Pleiotropic Effects in Light‐Related Skin Conditions, Photoaging and Skin Cancers, a Review,” International Journal of Molecular Sciences 17, no. 7 (2016): 1026. 27367679 10.3390/ijms17071026 PMC4964402

[17] R. Sarkar , E. B. Handog , A. Das , et al., “Topical and Systemic Therapies in Melasma: A Systematic Review,” Indian Dermatology Online Journal 14, no. 6 (2023): 769–781. 38099013 10.4103/idoj.idoj_490_22 PMC10718129

[18] A. G. Pandya , L. S. Hynan , R. Bhore , et al., “Reliability Assessment and Validation of the Melasma Area and Severity Index (MASI) and a New Modified MASI Scoring Method,” Journal of the American Academy of Dermatology 64, no. 1 (2011): 78–83.e2. 20398960 10.1016/j.jaad.2009.10.051

[19] C. Ruini , S. Schuh , E. Sattler , and J. Welzel , “Line‐Field Confocal Optical Coherence Tomography—Practical Applications in Dermatology and Comparison With Established Imaging Methods,” Skin Research and Technology 27, no. 3 (2021): 340–352. 33085784 10.1111/srt.12949

[20] A. C. Handel , L. D. B. Miot , and H. A. Miot , “Melasma: A Clinical and Epidemiological Review,” Anais Brasileiros de Dermatologia 89 (2014): 771–782. 25184917 10.1590/abd1806-4841.20143063 PMC4155956

[21] A. Achar and S. K. Rathi , “Melasma: A Clinico‐Epidemiological Study of 312 Cases,” Indian Journal of Dermatology 56, no. 4 (2011): 380–382. 21965843 10.4103/0019-5154.84722 PMC3178998

[22] M. Rendon , M. Berneburg , I. Arellano , and M. Picardo , “Treatment of Melasma,” Journal of the American Academy of Dermatology 54, no. 5 (2006): S272–S281. 16631968 10.1016/j.jaad.2005.12.039

[23] A. Banstola and X.‐L. Li , “Melasma Management: A Review of Current Treatment,” Nepal Journal of Dermatology, Venereology & Leprology 23, no. 2 (2025): 54–57.

[24] F. Prignano , J.‐P. Ortonne , G. Buggiani , and T. Lotti , “Therapeutical Approaches in Melasma,” Dermatologic Clinics 25, no. 3 (2007): 337–342. 17662899 10.1016/j.det.2007.04.006

[25] M. A. Middelkamp‐Hup , M. A. Pathak , C. Parrado , et al., “Oral Polypodium Leucotomos Extract Decreases Ultraviolet‐Induced Damage of Human Skin,” Journal of the American Academy of Dermatology 51, no. 6 (2004): 910–918. 15583582 10.1016/j.jaad.2004.06.027

[26] M. A. Middelkamp‐Hup , M. A. Pathak , C. Parrado , et al., “Orally Administered Polypodium Leucotomos Extract Decreases Psoralen‐UVA–Induced Phototoxicity, Pigmentation, and Damage of Human Skin,” Journal of the American Academy of Dermatology 50, no. 1 (2004): 41–49. 14699363 10.1016/s0190-9622(03)02732-4

[27] S. Gonzalez , Y. Gilaberte , N. Philips , and A. Juarranz , “Fernblock, a Nutriceutical With Photoprotective Properties and Potential Preventive Agent for Skin Photoaging and Photoinduced Skin Cancers,” International Journal of Molecular Sciences 12, no. 12 (2011): 8466–8475. 22272084 10.3390/ijms12128466 PMC3257081

[28] C.‐L. Goh , S. Y. Chuah , S. Tien , G. Thng , M. A. Vitale , and A. Delgado‐Rubin , “Double‐Blind, Placebo‐Controlled Trial to Evaluate the Effectiveness of Polypodium Leucotomos Extract in the Treatment of Melasma in Asian Skin: A Pilot Study,” Journal of Clinical and Aesthetic Dermatology 11, no. 3 (2018): 14–19. PMC5868779 29606995

[29] C. Marques , F. Hadjab , A. Porcello , et al., “Mechanistic Insights Into the Multiple Functions of Niacinamide: Therapeutic Implications and Cosmeceutical Applications in Functional Skincare Products,” Antioxidants 13, no. 4 (2024): 425. 38671873 10.3390/antiox13040425 PMC11047333

[30] A. G. Pedroso , G. R. D. Furtado , and K. L. Barbosa , “Niacinamide for the Treatment of Melasma: An Integrative Review of Randomized Clinical Trials,” Research, Society and Development 11, no. 11 (2022): 33581.

[31] H. L. Yang , C. P. Lin , Y. Vudhya Gowrisankar , et al., “The Anti‐Melanogenic Effects of Ellagic Acid Through Induction of Autophagy in Melanocytes and Suppression of UVA‐Activated α‐MSH Pathways via Nrf2 Activation in Keratinocytes,” Biochemical Pharmacology 185 (2021): 114454. 33545118 10.1016/j.bcp.2021.114454

[32] S. K. Rai , J. Yazdany , P. R. Fortin , and J. A. Aviña‐Zubieta , “Approaches for Estimating Minimal Clinically Important Differences in Systemic Lupus Erythematosus,” Arthritis Research & Therapy 17, no. 1 (2015): 143. 26036334 10.1186/s13075-015-0658-6 PMC4453215

